# Comparison of the expression of cytokine genes in the bursal tissues of the chickens following challenge with infectious bursal disease viruses of varying virulence

**DOI:** 10.1186/1743-422X-7-364

**Published:** 2010-12-08

**Authors:** Haiwen Liu, Manfu Zhang, Haitang Han, Jihong Yuan, Zandong Li

**Affiliations:** 1 State key Laboratory for Agrobiotechnology, College of Biological Sciences, China Agricultural University, No.2 Yuan Ming Yuan West Road, Beijing, 100193, China

## Abstract

**Background:**

Cytokines are important mediators and regulators of host responses against foreign antigen, with their main function to orchestrate the functional activities of the cells of the immune system. However little is known about the role of cytokines in pathogenesis and immune responses caused by infectious bursa disease virus (IBDV). The aim of this study was to examine the transcripts of cell-mediated immune response-related cytokine genes in the bursal tissues of chickens infected with IBDVs of varying virulence to gain an understanding of pathological changes and mechanisms of immunosuppression caused by IBDV infection and the immune responses evoked.

**Results:**

Real-time quantitative PCR analysis revealed that the expression levels of both Th1 [interferon (IFN)-γ, interleukins (IL)-2 and IL-12p40] and Th2 (IL-4, IL-5, IL-13 and IL-10) cytokines were significantly up-regulated following challenge with the H strain (vvIBDV) and up to 2- and 30-fold, respectively (*P *< 0.05). Following infection with the Ts strain (cell-adapted virus) these cytokine transcripts were up-regulated at 5 days post-infection (dpi), 2- and 13-fold respectively (*P *< 0.05), while the expression levels of IL-2 and IL-4 were not significantly different (*P *> 0.05). A higher degree of cytokine expression was induced by the H strain compared with the Ts strain.

**Conclusion:**

The results indicate that the expression of cell-mediated immune-related cytokine genes is strongly induced by IBDV, especially by the vvIBDV, H strain and reveal that these cytokines could play a crucial role in driving cellular immune responses during the acute phase of IBDV infection, and the cellular immune responses caused by IBDV of varying virulence are through different signaling pathways.

## Background

Infectious bursal disease (IBD), caused by infectious bursal disease virus (IBDV), is an acute, highly contagious and immunosuppressive disease in young chickens, resulting in great economic loss in the poultry industry [1]. IBDV can be differentiated into two serotypes (serotype 1 and 2) [2]. Serotype 1 shows different degrees of pathogenicity and mortality in chickens, whereas serotype 2 is avirulent in chickens [3]. Based on virulence, serotype 1 strains are classified as classically, intermediate, very or hypervirulent virulent [1].

IBDV is a non-enveloped, double-stranded (ds) RNA virus consisting of two segments, segment A (3.2 kb) and B (2.9 kb), encoding five proteins and belongs to the *Birnaviridae *family [4-6]. IBDV mainly affects young chickens from 3-6 weeks of age [7]. Although viral antigen has been detected in other organs within the first few hours of infection, the most extensive virus replication takes place primarily in the bursa of Fabricius [6]. Activated dividing B lymphocytes that secrete IgM^+ ^serve as target cells for the virus [8,9]. Viral infection results in lymphoid depletion of B cells and the destruction of bursal tissues [10], leading to an increased susceptibility to other infectious diseases and poor immune response to vaccines [5].

Replication of IBDV in the bursa is accompanied by an influx of T cells [8,11]. The marked influx of T cells into the infected bursa indicates that cell-mediated immunity plays important roles in the clearance of virus particles [12,13]. The T cells in the bursa of chickens infected by virus are activated, with up-regulated expression of a number of cytokine genes, such as interleukin (IL)-1β, IL-6 and interferon (IFN)-γ [14]. The change in the level of cytokine expression is closely associated with organizational destruction, inflammation and apoptosis [13]. The direct immunosuppressive effects of IBDV on T cells and their function remain unclear. Chickens infected with IBDV resulted in suppression of cellular immune responses and a subsequently reduction in the ability to respond to secondary infections [15].

The CD4^+ ^helper T (Th) cells play crucial roles in immune responses. The CD4^+ ^T cells have been classified as either Th1 or Th2 based on their cytokine profiles [16]. Th1 cells have evolved to enhance clearance of intracellular pathogens and are defined on the basis of their production of IFN-γ [17]. Th2 cells are critical for the control of certain parasitic infections through the production of the clustered group of cytokines IL-4, IL-5 and IL-13 [18]. Chickens are able to mount a typical Th1 or Th2 biased cytokine response after experimental viral and helminth parasitic infections, respectively [19]. Cytokines are important mediators and regulators of both types of host responses. However, little is known about the role of cytokine following IBDV infection. It is extremely important to gain an understanding of pathological changes and immunosuppression caused by IBDV infection and the immune responses evoked.

In the present study, our objective was to further investigate the Th1/Th2 paradigm by examining the transcriptional profile of cytokines in the bursal tissues of chickens infected with either vvIBDV H strain or the cell-adapted virus Ts strain at 1, 3 and 5 days post-infection (dpi) and also to test the hypothesis that the IBDVs with varying virulence induces different cytokine profiles during the course of infection.

## Results

### Generation of standard curves for real-time PCR analysis

Standard curves for the genes encoding IFN-γ, IL-2, IL-12p40, IL-4, IL-5, IL-13, IL-10, IBDV (H strain and Ts strain) and GAPDH were generated to determine relative quantification of cytokine expression and viral load in the bursa, with GAPDH used as the reference gene. A linear relationship was observed between the amount of input plasmid DNA and the C_t _values for the cytokines, reference gene and IBDV-specific products over six log10 dilutions. The equation for the standard curve and correlation coefficient (*r*^2^) for cytokines, IBDV and GAPDH are given in Table 1.

**Table 1:** Standard curve data from real-time PCR

Genes	Equation of standard curve	Correlation coefficient (*r*^2^)
GAPDH	Y = -3.48X + 39.02	0.997
IFN-γ	Y = -3.58X + 39.08	0.994
IL-2	Y = -3.62X + 37.54	0.994
IL-12P40	Y = -3.36X + 36.05	0.995
IL-4	Y = -3.16X + 39.42	0.991
IL-5	Y = -3.74X + 40.46	0.995
IL-13	Y = -4.02X + 40.87	0.991
IL-10	Y = -3.53X + 37.18	0.991
H strain Ts strain	Y = -3.50X + 39.94 Y = -3.60X + 40.50	0.992 0.994

### Changes in IBDV load in the bursa of Fabricius during the course of infection

**Figure 1:**
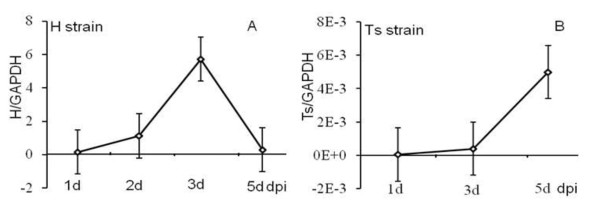
**Changes in virus load in the bursa tissues of chickens infected with either H or Ts strain**. Changes of IBDV load in the bursa were quantified by real-time PCR and presented as ratios of IBDV/GAPDH mRNA. The means and standard errors (SE) are from three separate experiments. dpi: days past-infection. A: H strain; B: Ts strain.

After infection with either H strain or Ts strain, viral load increased, reaching a maximum at 3 dpi in the H strain-infected birds (Figure 1A) and at 5 dpi in the Ts infected birds (Figure 1B). After peaking viral load was decreased significantly in the H and Ts-infected birds. Furthermore, viral load in the bursal tissues was higher at all time points in birds challenged with the H strain as compared with the Ts strain.

### Th1-cytokines expression during IBDV infection

**Figure 2:**
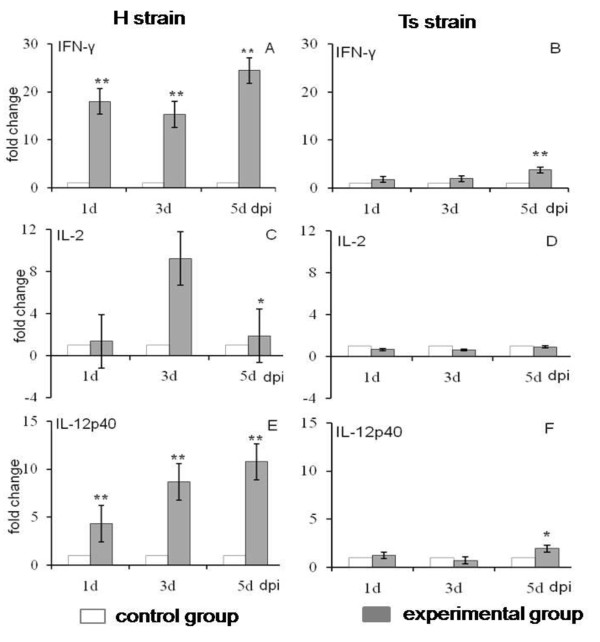
**Changes in Th1 cytokine expression in the bursa tissues of chickens infected with either H or Ts strain**. Changes in IFN-γ, IL-2 and IL-12p40 mRNA expression were quantified by real-time PCR and expressed as the fold-change in birds infected with either H or Ts strain of IBDV, when compared with uninfected birds. Bars show the means and standard errors (SE) from three separate experiments. The difference in cytokine expression between experimental and control was assessed by student's *t*-test and comparisons were considered significantly different at *P *≤ 0.05 (*) and at *P *< 0.01 (**). dpi: days past-infection.

Infection with IBDV resulted in transcriptional changes of mRNA encoding IFN-γ, IL-2 and IL-12p40 during the acute phase of the disease. Differences in cytokine expression were given as fold-change using the chicken GAPDH gene for normalization. Figure 2 shows the relative fold-change for the examined genes (IFN-γ, IL-2 and IL-12p40) in IBDV infected birds compared with uninfected birds. following infection with the H strain, the expression levels of IFN-γ and IL-12p40 genes (Figure 2A and 2E) in bursal tissues were significantly up-regulated compared with uninfected birds (*P *< 0.05) and the expression of IL-2 genes was markedly increased at 3 dpi with no differences at 1 and 5 dpi (*P *> 0.05) (Figure 2C). Furthermore the fold-change of the IFN-γ gene expression was the highest among the three cytokines. After infection with the Ts strain, the expression levels of IFN-γ and IL-12p40 genes in the bursa were not significantly different at 1 and 3 dpi (*P *> 0.05 for both genes at both time points), but then increased significantly at 5 dpi (*P *= 0.006 for IFN-γ and 0.02 for IL-12p40) (Figure 2B and 2F). However there was a slight downward trend of IL-2 expression during the course of the Ts infection (*P *> 0.05) (Figure 2D).

### Th2-cytokine expression during IBDV infection

**Figure 3:**
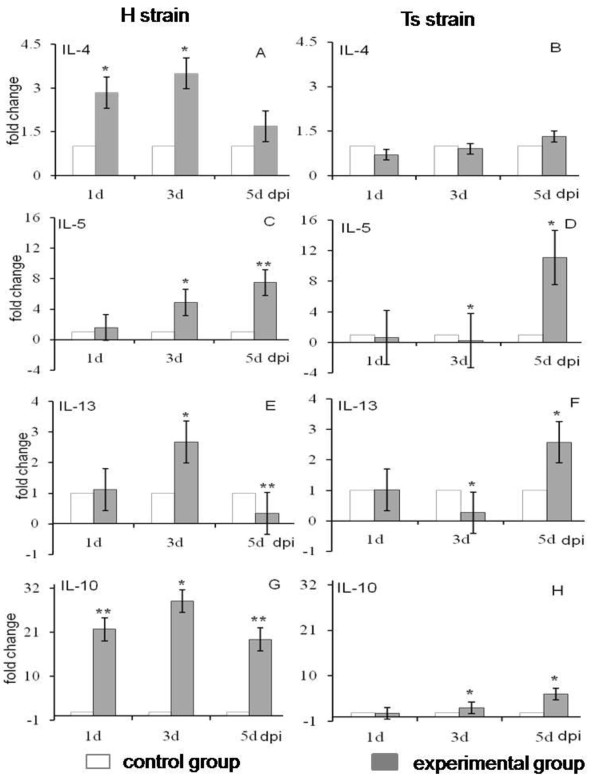
**Changes in Th2 cytokine expression in the bursa tissues of chickens infected with either H or Ts strain**. Changes in IL-4, IL-5, IL-13 and IL-10 mRNA expression were quantified by real-time PCR and expressed as fold-change in the birds infected with the H or Ts strain of IBDV, compared with uninfected birds. Bars show the means and standard errors (SE) from three separate experiments. The difference in cytokine expression between experimental group and control group was assessed by Student's *t*-test and comparisons were considered significantly different at *P *≤ 0.05 (*) and at *P *< 0.01 (**). dpi: days past-infection.

Temporal expression patterns of IL-4, IL-5, IL-13 and IL-10 genes in the bursa of chickens infected with the H or Ts strains are illustrated in Figure 3A-H. After H strain infection, the expression levels of IL-4, IL-13 and IL-10 in the bursa of chickens were up-regulated and peaked at 3 dpi and then declined at 5 dpi (Figure 3A, 3E and 3G). The change in expression of IL-10 at 3 dpi was 28.8-fold higher (*P *= 0.03). In contrast, the expression of IL-5 mRNA in the bursa of birds infected with the H strain increased continuously, peaking at 5 dpi with a 7.47-fold increase (*P *= 0.00002) (Figure 3C). The expression of the IL-4 gene in the bursa was not significantly different (*P *> 0.05) between the Ts-infected and control group (Figure 3B). After Ts infection, the expression pattern of the IL-5 gene was similar to that of IL-13, but not significantly different at 1 dpi (*P *> 0.05), then obviously down-regulated at 3 dpi (*P *< 0.05), but significantly up-regulated at 5 dpi (*P *< 0.05) compared with control birds (Figure 3D and 3F). The expression of the IL-10 gene in the bursa of chickens infected with the Ts strain was lightly up-regulated at 1 and 3 dpi (*P *> 0.05), and then was increased significantly at 5 dpi compared with the control group (*P *= 0.02) (Figure 3H).

## Discussion

Avian cytokines, like their mammalian counterparts, are influential in host immune response to pathogenic infection [20]. The cytokine responses to IBDV in the bursa of chickens are poorly described and their role in the pathogenesis of such infections has not yet been extensively studied. Of the seven genes examined in this study, the levels of expression of IFN-γ, IL-2 and IL-12P40 genes in the bursa tissues following H strain infection were increased compared with the IL-4, IL-5, IL-13 and IL-10 genes; the expression levels of IL-4, IL-5, IL-13 and IL-10 genes in Ts-infected chicken bursa had a higher fold-change than the IFN-γ, IL-2 and IL-12p40 genes. The results obtained from gene expression analysis of Th1 and Th2 cytokines revealed that the vvIBDV, H strain induces an immune response characteristic of the Th1 pathway; In contrast, the cell-adapted virus, Ts strain induced an immune response characteristic of the Th2 pathway. The results also revealed the early activation of a variety of antiviral host defenses after infection, for example activation of innate immune responses, cell-mediated immune responses and modulation of host transcription.

Our results showed that the viral load in bursal tissues increased approximately 1000-fold after infection with the H strain compared with the Ts strain (Figure 1). The vvIBDV, H strain had a stronger capability of replication and spread in the bursa of birds than cell-adapted Ts strain. Based on earlier work from our laboratory [21], the doses used in this study contained 10^3.4 ^egg infectious dose_50 _(EID_50_) H strain and 10^6.5 ^tissue culture infectious dose_50 _(TCID_50_) Ts strain and should have demonstrated a positive signal at approximately the bursal tissues the same time. These date indicate that the load and replication of IBDV in the bursa is closely related to clinical symptoms and pathology, which is in accordance with Eldaghayes's study [14].

The present studies showed that there was a trend of up- or down-regulation in the expression levels of several cytokine genes in the bursa following either H strain or Ts strain infection. A higher viral load in the bursa was associated with significantly higher expression of cytokine genes. This is in agreement with the previous reports made by Abel and Abdul-Careem [22,23] who studied virus replication and cytokine gene expression following virus infection and found a significant association between higher viral RNA levels and cytokine transcript concentration in various tissues. These results demonstrate that the difference in the expression levels of cytokines was possibly influenced by the different degree of viral replication. However, factors influencing the timing of cytokine regulation in bursal tissues and the cause and effect relationship between host response and viral replication are not clear from the present observations. Future experiments need to be conducted to examine more cytokines during the course of infection with variously virulent IBDVs.

Our results suggest that the H strain tends to up-regulate the Th1 cytokines response. Th1 cells are characterized by the secretion of IFN-γ, IL-2 and IL-12p40, and a strong cell-mediated immunity that is geared towards effective elimination of intracellular pathogens such as viruses [17]. IL-2 stimulates proliferation of chicken T lymphocytes and NK cells [24-26]. Production of IL-12 and IFN-γ is critical to host defense against intracellular pathogens [27], indicating that it is possible to observe simultaneous up-regulation of IFN-γ and IL-10 in response to IBDV infection. The observed increase in IFN-γ expression in IBDV-infected bursa presumably reflects the inflammatory response and is consistent with earlier published results [14,28], suggesting that cell-mediated responses are initiated to resolve infections. IFN-γ-induced activation of macrophages (Mφ) results in the stimulation of nitric oxide synthase (iNOS), which in turn leads to the production of apoptotic mediators such as nitric oxide (NO) or tumor necrosis factor-α (TNF-α) [29]. Previous studies from our laboratory demonstrated that apoptosis was induced by the vvIBDV H strain in the chicken bursa [30]. Furthermore our observations suggest that enhanced IFN-γ expression was associated with disease progression in IBDV- infected chickens.

However the Ts strain tends to up-regulate effects of the Th2 cytokine response. Th2 cells secrete IL-4, IL-5, IL-13 and IL-10, and are geared towards a humoral immune response against parasites and allergic reactions [18]. IL-4 has been shown to direct B cells to produce the anti-allergen IgE, to inhibit Th1 cell function and to prevent the production of IL-2, IL-12 and IFN-γ that are necessary for development of cytotoxic T cells [31]. However our results did not observe that up-regulation in the expression of Th2 cytokines suppressed transcriptional activities of Th1 cytokines. Previous reports by Heidari [32] have shown that because of the substantial level of IL-4 mRNA expression in the Marek's disease virus (MDV)-infected birds. It is not unusual to observe transcriptional activities of IL-2 being severely suppressed. A change in cytokine expression levels is closely related to virulence and replication of IBDV, but the mechanism by which this occurs is still not fully understood.

As expected, IBDV infection caused cell-mediated immune-related cytokine responses in the bursa, as shown (Figure 2 and 3) by the up-regulation or down-regulation of detected cytokines expression. This is in accordance with the pathology and clinical signs of two distinctly virulent IBDV. Infection with vvIBDV results in lymphoid depletion, marked atrophy of the bursal tissues and high rates of mortality [33,34], but Ts strain infection does not cause obviously clinical signs [21].

Of the evaluated cytokines, prolonged expression of IFN-γ and IL-10 genes was up-regulated due to IBDV infection. IL-10 is a potent stimulator of NK cells [35,36], a function that might contribute to the clearance of the pathogen and facilitate antigen acquisition from dead cells for cross-priming activated antigen-presenting cells (APCs), providing a link between the innate and the adaptive immune responses [37]. The expression of IL-10 in the bursa following IBDV infection has not been studied previously. In the present study our results indicated that IL-10 expression was markedly increased and similar to the extent of up-regulated expression of IFN-γ following infection by the H or Ts strain. This is consistent with the fact that IL-10 plays a dual role in infectious diseases [37] and is in agreement with the observation made recently by Abdul-Careem [22] who recorded that the expression of the IL-10 gene followed the pattern of expression of the IFN-γ gene to a certain extent in both pre- and post-hatched herpesvirus of turkey (HVT)-immunized chickens. In general, both IL-10 and IFN-γ are known to be important cytokines in the cell-mediated immune response and evoke host responses to the pathogen in chickens [38,39]. This also suggests that IL-10 may play a role as an immunostimulatory cytokine similar to IFN-γ after IBDV infection.

## Conclusions

In summary, we have shown that infection with IBDV induces changes in the level of expression related to Th1 and Th2 in the chicken bursa and that the vvIBDV, H strain strongly induced an increase in cytokine expression. It is clear that changes in the extent of cytokine expression were closely associated with virulence of the virus and viral replication. Further studies are necessary to elucidate the function of the cytokines in pathogenesis and immunity against IBDV.

## Materials and methods

### Chickens and virus

Four-week-old specific pathogen-free (SPF) white leghorn chickens purchased from Meria (Meria, Beijing, China) were housed in isolators with water and food freely available.

The H strain (vvIBDV) [21,34] was provided by the Harbin Veterinary Research Institute of the Chinese Academy of Agricultural Sciences. When SPF chickens were inoculated with the H strain at a dose of 2 × 10^3 ^egg infectious dose_50 _(EID_50_), 60% mortality resulted. The Ts strain, a cell-adapted virus supplied by our laboratory [21] resulted in 0% mortality and was used as a reference moderately virulent strain. The virus was propagated and the titers of both virus stocks were determined as previously described [21,40]. The stock of the H strain was 10^3.4 ^EID_50 _per 0.2 ml and was used as an inoculums following 20-fold dilution. The tissue culture infectious dose_50 _(TCID_50_) of the Ts strain was 10^6.5 ^per 0.1 ml and was diluted 200-fold and used as an inoculum.

### Virus infection and collection of bursa samples

Four-week-old SPF chickens were randomly divided into three groups and housed in three isolators under the same conditions. Groups 1 (n = 25) and 2 (n = 15) were infected respectively with either the H or Ts strain by the eyes and nose-drop routes. Each bird was inoculated with 0.2 ml of virus dilution. Chickens in group 3 (n = 12) were inoculated with 0.2 ml of phosphate-buffered saline (PBS) per bird to serve as controls. At 1, 3 and 5dpi the bursal tissues (n = 3) were collected separately from the infected and control groups, placed immediately in liquid nitrogen and stored at -80°C until further required.

### Extraction of total RNA and cDNA synthesis

Total RNA was isolated from bursal tissues using a total RNA extraction kit (Tiangen, BeiJing, China) according to the manufacturer's instructions and eluted into a 60 μl volume of diethylpyrocarbonate (DEPC)-treated water. To eliminate possible contamination with genomic DNA, 0.1 U/μl DNase (Promega, Madison, WI, USA) was applied according to the manufacturer's protocol. A reverse transcription reaction of total RNA (1 μl) was carried out to synthesize cDNA using an iScript™cDNA Synthesis kit (Promega) following the manufacturer's instructions with minor modifications. First, 1 μg of total RNA, 1 μl of random hexamer primers and DEPC-treated water was denatured at 95°C for 10 min, then chilled on ice for 5 min. The RNA was mixed with a previously prepared mixture in a final volume of 20 μl, and left at room temperature for 10 min. the mixture was then incubated at 42°C for 60 min to synthesize cDNA and heated at 95°C for 5 min to inactivate the reverse transcriptase. The synthetic cDNA was stored at -20°C.

### Primers

Primers were designed corresponding to sequences from GenBank using Applied Biosystems pring express software v3.0 (Applied Biosystems, Carlsbad, CA). The primers were synthesized by Sangon (Sangon, Beijing, China). Previously published primers for IFN-γ and GAPDH [32] were used in the present study and GAPDH was used as the reference gene (Table 2). The specificity for each primer set was tested by analyzing the melting curve following real-time PCR.

**Table 2:** Sequence of the primers used in real-time PCR

Genes	Direction	Sequence	Product (bp)	Accession no. in GenBank
GAPDH^a^	Forward	TGCCATCACAGCCACACAGAAG	123	AF047874.1
	Reverse	ACTTTCCCCACAGCCTTAGCAG		
IFN-γ^a^	Forward	AAGTCAAAGCCGCACATCAAAC	132	X99774.1
	Reverse	CTGGATTCTCAAGTCGTTCATCG		
IL-2	Forward	TTCTGGGACCACTGTATGCTCTT	129	AF000631.1
	Reverse	TACCGACAAAGTGAGAATCAATCAG		
IL-12P40	Forward	CGAAGTGAAGGAGTTCCCAGAT	123	AY262752.1
	Reverse	GACCGTATCATTTGCCCATTG		
IL-4	Forward	AATGACATCCAGGGAGAGGTTTC	100	AJ621249.1
	Reverse	AGGCTTTGCATAAGAGCTCAGTTT		
IL-5	Forward	GGAACGGCACTGTTGAAAAATAA	111	AJ621252.1
	Reverse	TTCTCCCTCTCCTGTCAGTTGTG		
IL-13	Forward	CTGCCCTTGCTCTCCTCTGT	123	AJ621250.1
	Reverse	CCTGCACTCCTCTGTTGAGCTT		
IL-10	Forward	GCTGAGGGTGAAGTTTGAGGAA	142	AF000631.1
	Reverse	GAAGCGCAGCATCTCTGACA		
H strain	Forward	CACTCCCTGGTGGCGTTTA	126	AY321955.1
	Forward	TGTCGTTGATGTTGGCTGTTG		
Ts strain	Forward	ACCGGCACCGACAACCTTA	117	AF076230.1
	Reverse	CCCTGCCTGACCACCACTT		

^a^Primer sequences from reference 32.

### Preparation of standard curves

The standard curves of all genes detected in this study were made with appropriate modifications as previously described [41-43]. For the preparation of the standards curves, all genes were amplified with the following cycling parameters: pre-incubation and denaturation at 95°C for 5 min, followed by 30 cycles of 95°C for 30 s, 60°C for 30 s, and 72°C for 30 s. A final extension step was carried out at 72°C for 5 min. The PCR products were then eluted from the Agarose gel and linked into the pEGM-T easy vector (Promega) and transformed into competent DH5α *Escherichia coli *cells (Takara Bio Inc, Japan) according to the manufacturer's instructions. The identified positive clones were grown and plasmid DNA isolated using a miniprep kit from Axygen (Axygen, CA, USA). Subsequently, 10-fold serial dilutions (10^-1^-10^-6^) of the plasmid DNA stocks were made and assayed in triplicate by real-time PCR to generate standard curves for quantification by Applied Biosystems SDS 2.2. The correlation coefficient of standard curves exceeded or equaled 0.99 (Table 1).

### Real-time PCR and data Processing

The analysis of real-time PCR data and relative quantification of cytokines and IBDV genes was carried out by the 7900HT Sequence Detection System (Applied Biosystems). The PCR was performed in a 20 μl volume containing 1 μl cDNA, 10 μl 2 × power SYBR Green PCR master mix (Applied Biosystems, Forster City, CA), 300 nM of each gene-specific primer. Thermal cycling parameters were as follows: 50°C for 2 min, 95°C for 10 min, 40 cycles of 95°C for 15 s and 60°C for 1 min, followed by one cycle of 95°C for 15 s, 60°C for 15 s and 95°C for 15 s. The final step was to obtain a melt curve for the PCR products to determine the specificity of amplification. All standard dilutions, controls and infected samples were carried out in triplicate on the same plate, and each reaction plate contained two standard curves for both target and reference genes in the same preparation. Furthermore, triplicate samples were assayed for each experiment and GAPDH was utilized as the reference gene.

The quantification of cytokine gene expression by real-time PCR was conducted as detailed elsewhere [42,44]. Expression levels of cytokine genes were calculated relative to the expression of the GAPDH gene and expressed as an n-fold increase or decrease relative to the control samples.

### Statistical analysis

All date analyses were performed using Microsoft^® ^Excel 2007. Student's *t*-test was used to detect significant differences between infected and control groups. A *P***-**value ≤ 0.05 was considered significant.

## Competing interests

The authors declare that they have no competing interests.

## Authors' contributions

HWL carried out all the experiments, analyzed results and drafted the manuscript. HTH helped to edit the manuscript. Some help was given by JHY in analysis of data and preparation of the manuscript. MFZ and ZDL participated in the design of the study and the critical view of manuscript writing. All authors read and approved the final manuscript.

## References

[B1] van den Berg TP Eterradossi N Toquin D Meulemans G Infectious bursal disease (Gumboro disease) Rev Sci Tech 2000 19 509 543 10935278

[B2] McFerran JB McNulty MS McKillop ER Connor TJ McCracken RM Collins DS Allan GM Isolation and serological studies with infectious bursal disease viruses from fowl, turkeys and ducks: demonstration of a second serotype Avian Pathol 1980 9 395 404 10.1080/03079458008418423 18770277

[B3] Ismail NM Saif YM Moorhead PD Lack of pathogenicity of five serotype 2 infectious bursal disease viruses in chickens Avian Dis 1988 32 757 759 10.2307/1590995 2849404

[B4] Mundt E Kollner B Kretzschmar D VP5 of infectious bursal disease virus is not essential for viral replication in cell culture J Virol 1997 71 5647 5651 9188642 10.1128/jvi.71.7.5647-5651.1997 PMC191810

[B5] Kibenge FS Dhillon AS Russell RG Biochemistry and immunology of infectious bursal disease virus J Gen Virol 1988 69 Pt 8 1757 1775 10.1099/0022-1317-69-8-1757 2841403

[B6] Dobos P Hill BJ Hallett R Kells DT Becht H Teninges D Biophysical and biochemical characterization of five animal viruses with bisegmented double-stranded RNA genomes J Virol 1979 32 593 605 228080 10.1128/jvi.32.2.593-605.1979 PMC353591

[B7] Hoffmann-Fezer G Lade R [Post-hatching development and involution of the Bursa Fabricii in the chicken (Gallus domesticus)] Z Zellforsch Mikrosk Anat 1972 124 406 418 10.1007/BF00355039 5011359

[B8] Sharma JM Kim IJ Rautenschlein S Yeh HY Infectious bursal disease virus of chickens: pathogenesis and immunosuppression Dev Comp Immunol 2000 24 223 235 10.1016/S0145-305X(99)00074-9 10717289

[B9] Hirai K Calnek BW In vitro replication of infectious bursal disease virus in established lymphoid cell lines and chicken B lymphocytes Infect Immun 1979 25 964 970 227789 10.1128/iai.25.3.964-970.1979 PMC414542

[B10] Kaufer I Weiss E Significance of bursa of Fabricius as target organ in infectious bursal disease of chickens Infect Immun 1980 27 364 367 6247275 10.1128/iai.27.2.364-367.1980 PMC550773

[B11] Kim IJ You SK Kim H Yeh HY Sharma JM Characteristics of bursal T lymphocytes induced by infectious bursal disease virus J Virol 2000 74 8884 8892 10.1128/JVI.74.19.8884-8892.2000 10982331 PMC102083

[B12] Williams AE Davison TF Enhanced immunopathology induced by very virulent infectious bursal disease virus Avian Pathol 2005 34 4 14 10.1080/03079450400025364 15763733

[B13] Rautenschlein S Yeh HY Njenga MK Sharma JM Role of intrabursal T cells in infectious bursal disease virus (IBDV) infection: T cells promote viral clearance but delay follicular recovery Arch Virol 2002 147 285 304 10.1007/s705-002-8320-2 11890524

[B14] Eldaghayes I Rothwell L Williams A Withers D Balu S Davison F Kaiser P Infectious bursal disease virus: strains that differ in virulence differentially modulate the innate immune response to infection in the chicken bursa Viral Immunol 2006 19 83 91 10.1089/vim.2006.19.83 16553553

[B15] Kim IJ Karaca K Pertile TL Erickson SA Sharma JM Enhanced expression of cytokine genes in spleen macrophages during acute infection with infectious bursal disease virus in chickens Vet Immunol Immunopathol 1998 61 331 341 10.1016/S0165-2427(97)00135-9 9613445

[B16] Janeway CJ The immune system evolved to discriminate infectious nonself from noninfectious self Immunol Today 1992 13 11 16 10.1016/0167-5699(92)90198-G 1739426

[B17] Kunzendorf U Tran TH Bulfone-Paus S The Th1-Th2 paradigm in 1998: law of nature or rule with exceptions Nephrol Dial Transplant 1998 13 2445 2448 10.1093/ndt/13.10.2445 9794533

[B18] Avery S Rothwell L Degen WD Schijns VE Young J Kaufman J Kaiser P Characterization of the first nonmammalian T2 cytokine gene cluster: the cluster contains functional single-copy genes for IL-3, IL-4, IL-13, and GM-CSF, a gene for IL-5 that appears to be a pseudogene, and a gene encoding another cytokinelike transcript, KK34 J Interferon Cytokine Res 2004 24 600 610 15626157 10.1089/jir.2004.24.600

[B19] Degen WG Daal N Rothwell L Kaiser P Schijns VE Th1/Th2 polarization by viral and helminth infection in birds Vet Microbiol 2005 105 163 167 10.1016/j.vetmic.2004.12.001 15708812

[B20] Kaiser Pete Stabeli Peter Fred Davison, Bernd Kaspers, Karel A Avian Cytokines and Chemokines: Avian Immunology 2008 Chapter 10 First Schat, Published by Elsevier Ltd 203 222

[B21] Zhang MF Huang GM Qiao S Early stages of infectious bursal disease virus infection in chickens detected by in situ reverse transcriptase-polymerase chain reaction Avian Pathol 2002 31 593 597 10.1080/0307945021000024580 12593743

[B22] Abdul-Careem MF Hunter DB Lambourne MD Read LR Parvizi P Sharif S Expression of cytokine genes following pre- and post-hatch immunization of chickens with herpesvirus of turkeys Vaccine 2008 26 2369 2377 10.1016/j.vaccine.2008.02.069 18406020

[B23] Abel K Rocke DM Chohan B Fritts L Miller CJ Temporal and anatomic relationship between virus replication and cytokine gene expression after vaginal simian immunodeficiency virus infection J Virol 2005 79 12164 12172 10.1128/JVI.79.19.12164-12172.2005 16160143 PMC1211549

[B24] Hilton LS Bean AG Kimpton WG Lowenthal JW Interleukin-2 directly induces activation and proliferation of chicken T cells in vivo J Interferon Cytokine Res 2002 22 755 763 10.1089/107999002320271341 12184913

[B25] Lillehoj HS Min W Choi KD Babu US Burnside J Miyamoto T Rosenthal BM Lillehoj EP Molecular, cellular, and functional characterization of chicken cytokines homologous to mammalian IL-15 and IL-2 Vet Immunol Immunopathol 2001 82 229 244 10.1016/S0165-2427(01)00360-9 11587737

[B26] Choi KD Lillehoj HS Role of chicken IL-2 on gammadelta T-cells and Eimeria acervulina-induced changes in intestinal IL-2 mRNA expression and gammadelta T-cells Vet Immunol Immunopathol 2000 73 309 321 10.1016/S0165-2427(00)00148-3 10713343

[B27] Jouanguy E Doffinger R Dupuis S Pallier A Altare F Casanova JL IL-12 and IFN-gamma in host defense against mycobacteria and salmonella in mice and men Curr Opin Immunol 1999 11 346 351 10.1016/S0952-7915(99)80055-7 10375558

[B28] Rautenschlein S Yeh HY Sharma JM Comparative immunopathogenesis of mild, intermediate, and virulent strains of classic infectious bursal disease virus Avian Dis 2003 47 66 78 10.1637/0005-2086(2003)047[0066:CIOMIA]2.0.CO;2 12713160

[B29] Lowenstein CJ Alley EW Raval P Snowman AM Snyder SH Russell SW Murphy WJ Macrophage nitric oxide synthase gene: two upstream regions mediate induction by interferon gamma and lipopolysaccharide Proc Natl Acad Sci USA 1993 90 9730 9734 10.1073/pnas.90.20.9730 7692452 PMC47644

[B30] Renmao HWAM Li Apoptosis Induction by the 5 NCR of Infectious Bursal Disease Virus The Open Veterinary Science Journal 2009 3 55 63

[B31] Becker Y The changes in the T helper 1 (Th1) and T helper 2 (Th2) cytokine balance during HIV-1 infection are indicative of an allergic response to viral proteins that may be reversed by Th2 cytokine inhibitors and immune response modifiers--a review and hypothesis Virus Genes 2004 28 5 18 10.1023/B:VIRU.0000012260.32578.72 14739648

[B32] Heidari M Zhang HM Sharif S Marek's disease virus induces Th-2 activity during cytolytic infection Viral Immunol 2008 21 203 214 10.1089/vim.2007.0078 18433333

[B33] Withers DR Young JR Davison TF Infectious bursal disease virus-induced immunosuppression in the chick is associated with the presence of undifferentiated follicles in the recovering bursa Viral Immunol 2005 18 127 137 10.1089/vim.2005.18.127 15802957

[B34] Xia RX Wang HY Huang GM Zhang MF Sequence and phylogenetic analysis of a Chinese very virulent infectious bursal disease virus Arch Virol 2008 153 1725 1729 10.1007/s00705-008-0140-8 18622570

[B35] Albert ML Sauter B Bhardwaj N Dendritic cells acquire antigen from apoptotic cells and induce class I-restricted CTLs Nature 1998 392 86 89 10.1038/32183 9510252

[B36] Salazar-Onfray F Petersson M Franksson L Matsuda M Blankenstein T Karre K Kiessling R IL-10 converts mouse lymphoma cells to a CTL-resistant, NK-sensitive phenotype with low but peptide-inducible MHC class I expression J Immunol 1995 154 6291 6298 7759867

[B37] Mocellin S Panelli MC Wang E Nagorsen D Marincola FM The dual role of IL-10 Trends Immunol 2003 24 36 43 10.1016/S1471-4906(02)00009-1 12495723

[B38] Abdul-Careem MF Hunter BD Parvizi P Haghighi HR Thanthrige-Don N Sharif S Cytokine gene expression patterns associated with immunization against Marek's disease in chickens Vaccine 2007 25 424 432 10.1016/j.vaccine.2006.08.006 17070626

[B39] Djeraba A Bernardet N Dambrine G Quere P Nitric oxide inhibits Marek's disease virus replication but is not the single decisive factor in interferon-gamma-mediated viral inhibition Virology 2000 277 58 65 10.1006/viro.2000.0576 11062036

[B40] Reed MH LJ A simple method of estimating fifty percent endpoints 1938 27 493 497

[B41] Abdul-Careem MF Hunter BD Nagy E Read LR Sanei B Spencer JL Sharif S Development of a real-time PCR assay using SYBR Green chemistry for monitoring Marek's disease virus genome load in feather tips J Virol Methods 2006 133 34 40 10.1016/j.jviromet.2005.10.018 16300836

[B42] Peirson SN Butler JN Foster RG Experimental validation of novel and conventional approaches to quantitative real-time PCR data analysis Nucleic Acids Res 2003 31 e73 10.1093/nar/gng073 12853650 PMC167648

[B43] Whelan JA Russell NB Whelan MA A method for the absolute quantification of cDNA using real-time PCR J Immunol Methods 2003 278 261 269 10.1016/S0022-1759(03)00223-0 12957413

[B44] Cikos S Bukovska A Koppel J Relative quantification of mRNA: comparison of methods currently used for real-time PCR data analysis BMC Mol Biol 2007 8 113 10.1186/1471-2199-8-113 18093344 PMC2235892

